# Prevalence and Genetic Characterization of Pertactin-Deficient *Bordetella pertussis* in Japan

**DOI:** 10.1371/journal.pone.0031985

**Published:** 2012-02-14

**Authors:** Nao Otsuka, Hyun-Ja Han, Hiromi Toyoizumi-Ajisaka, Yukitsugu Nakamura, Yoshichika Arakawa, Keigo Shibayama, Kazunari Kamachi

**Affiliations:** Department of Bacteriology II, National Institute of Infectious Diseases, Tokyo, Japan; Instituto Butantan, Brazil

## Abstract

The adhesin pertactin (Prn) is one of the major virulence factors of *Bordetella pertussis*, the etiological agent of whooping cough. However, a significant prevalence of Prn-deficient (Prn^−^) *B. pertussis* was observed in Japan. The Prn^−^ isolate was first discovered in 1997, and 33 (27%) Prn^−^ isolates were identified among 121 *B. pertussis* isolates collected from 1990 to 2009. Sequence analysis revealed that all the Prn^−^ isolates harbor exclusively the vaccine-type *prn1* allele and that loss of Prn expression is caused by 2 different mutations: an 84-bp deletion of the *prn* signal sequence (*prn1*ΔSS, *n* = 24) and an IS*481* insertion in *prn1* (*prn1*::IS*481*, *n* = 9). The frequency of Prn^−^ isolates, notably those harboring *prn1*ΔSS, significantly increased since the early 2000s, and Prn^−^ isolates were subsequently found nationwide. Multilocus variable-number tandem repeat analysis (MLVA) revealed that 24 (73%) of 33 Prn^−^ isolates belong to MLVA-186, and 6 and 3 Prn^−^ isolates belong to MLVA-194 and MLVA-226, respectively. The 3 MLVA types are phylogenetically closely related, suggesting that the 2 Prn^−^ clinical strains (harboring *prn1*ΔSS and *prn1*::IS*481*) have clonally expanded in Japan. Growth competition assays in vitro also demonstrated that Prn^−^ isolates have a higher growth potential than the Prn^+^ back-mutants from which they were derived. Our observations suggested that human host factors (genetic factors and immune status) that select for Prn^−^ strains have arisen and that Prn expression is not essential for fitness under these conditions.

## Introduction

*Bordetella pertussis* is the causative agent of pertussis or whooping cough, a highly contagious disease of the human upper respiratory tract. Adolescents and adults are its primary reservoir and play a crucial role in the transmission of the microbe to infants and unvaccinated children [1], [2]. Immunization is the most effective method for the prevention and control of pertussis. In Japan, acellular pertussis (aP) vaccines were introduced in 1981 and pertussis has been controlled by means of a schedule of three primary doses and a single booster dose at ages 3, 4, 5, and 18–23 months. The vaccine coverage with three primary does has been ≥90%.

*B. pertussis* produces several virulence factors that contribute to its adherence to the respiratory ciliate epithelium. The virulence factors pertussis toxin (PT) and filamentous haemagglutinin (FHA) are critical antigens responsible for inducing immunity to *B. pertussis* and are included as major antigens in aP vaccines. Some aP vaccines include either the virulence factor pertactin (Prn) and/or fimbriae (Fim) as additional antigen(s). Among four currently used Japanese aP vaccines, two vaccines contain Prn (5–7.5 µg per 0.5 ml dose) and Fim2 (1 µg/dose), and others do not contain both Prn and Fim2 [3]. In contrast, aP vaccines widely used in Europe and the USA contain from 3 to 8 µg per dose of Prn: Infanrix, 8 µg; DAPTACEL, 3 µg. The three-component aP vaccine containing PT, FHA, and Prn is more effective than the two-component aP vaccine consisting of only PT and FHA [4], [5]. In vaccine efficacy trials, the anti-Prn antibody level correlates with clinical protection, suggesting an important role for Prn in immunity [6]. In vitro studies also show that anti-Prn antibody is crucial for opsonophagocytosis [7].

Prn belongs to the type V autotransporter family whose members undergo autoproteolytic processing; mature Prn is a 69-kDa protein that is attached to the bacterial cell surface [8], [9], [10]. This protein contains an RGD (Arg-Gly-Asp) motif, which is implicated in ligand-receptor interactions in eukaryotes [11]. Prn is considered to function as an adhesin that can bind human epithelial cells; however, the host receptor for Prn has not been identified. Besides its potential role as an adhesin, *Bordetella bronchiseptica* Prn has been shown to function as a phage receptor [12], [13]. During the last decade, Prn polymorphism has been described among *B. pertussis* strains circulating worldwide. Prn variation is mainly limited to 2 regions, designated as region 1 (R1) and region 2 (R2), which are composed of the repeat motifs (GGXXP)n and (PQP)n, respectively [14]. Most variations are found in R1, which is located adjacent to an RGD motif. Thirteen Prn variants have been identified so far [15], [16]. In Japan, Prn1 and Prn2 variants currently predominate; however, the vaccine-type Prn1 has been gradually replaced with the nonvaccine-type Prn2 since the mid-1990s [17]. A recent study shows that the ability of *B. pertussis* strains to colonize mouse lung decreases in the order Prn1>Prn2 and Prn3 [18].

*B. pertussis* Prn^−^ isolates are present in Europe [19], [20]. The Prn^−^ isolates were collected in Italy (*n* = 1) and France (*n* = 4), and this phenotype is due to the deletion of *prn* or insertion of the IS*481* element. The IS*481* is present in multiple copies in the *B. pertussis* chromosome, causing frequent chromosomal rearrangements and deletions [21], [22]. The emerging Prn^−^ strains raise the possibility that the prevalence of Prn^−^ strains reduces the efficacy of aP vaccines containing Prn. Here, we identified the significant prevalence of Prn^−^ strains recently circulating in Japan. To obtain detailed insights into these strains with respect to their genetic, temporal, and geographical characteristics, we performed sequence analysis and multilocus variable-number tandem repeat analysis (MLVA). Using an in vitro growth competition assay, we attempted to gain insights into the biological mechanisms responsible for the prevalence of Prn^−^ strains.

## Results

### Identification of Prn^−^ isolates

**Figure 1:**
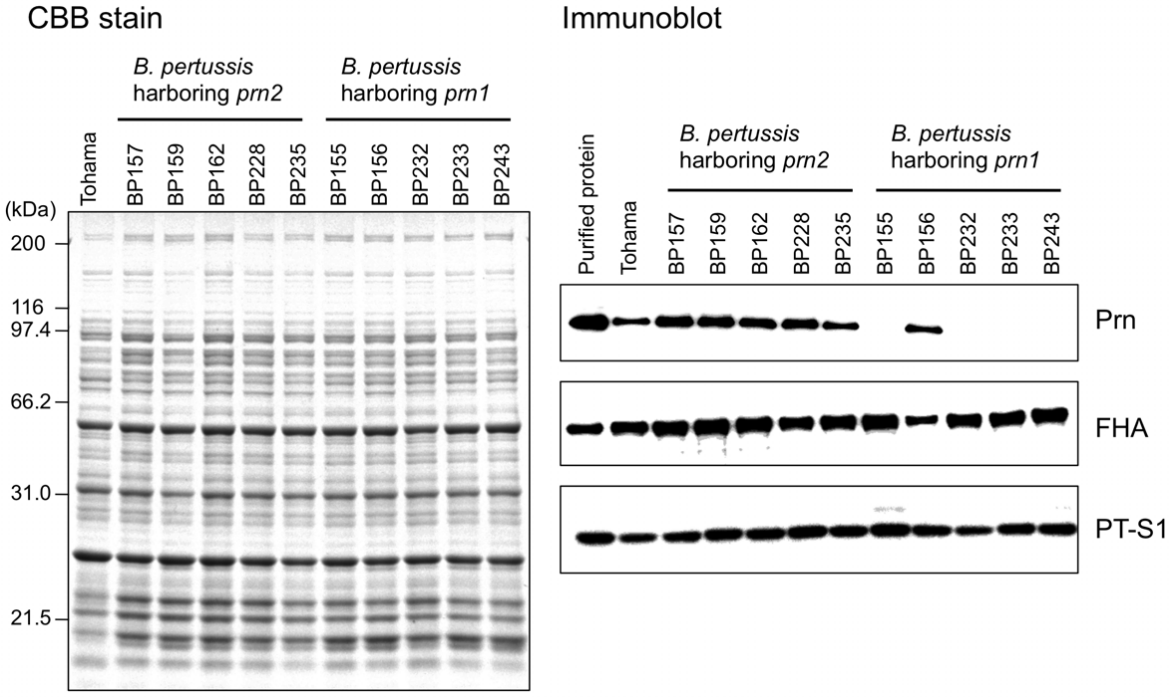
Prn expression in *B. pertussis* clinical isolates. The isolates harboring *prn2* allele (BP157, BP159, BP162, BP228, and BP235) and *prn1* allele (BP155, BP156, BP232, BP233, and BP243) were cultured on CSM plates. Total protein (10 µg) extracted from the bacterial cells was separated by SDS-PAGE followed by CBB R-250 staining (left panel). Immunoblots (1 µg protein/lane) were incubated with anti-Prn1, anti-PT or anti-FHA antiserum (right panel). Ten ng of purified Prn1, PT, or FHA and total protein (1 µg) from *B. pertussis* Tohama were run on the gel as positive controls.

*B. pertussis* Prn expression was analyzed by immunoblotting with anti-Prn1 antiserum. Figure 1 shows a representative blot of 6 Prn-positive and 4 negative isolates. Total 33 Prn^−^ isolates were identified among 121 *B. pertussis* isolates collected in 1990–2009 in Japan, which we acquired from the National Institute of Infectious Diseases (NIID), Japan. Interestingly, all Prn^−^ isolates harbor vaccine-type *prn1* and *ptxA2* alleles. The expression of other virulence factors PT, FHA, and Fim3 was detected in the recent Prn^−^ isolates (collected in 2005–2009) by immunoblotting and serotyping. Detailed information on these 121 isolates is listed in Table S1.

### Sequence analysis of Prn^−^ isolates

**Figure 2:**
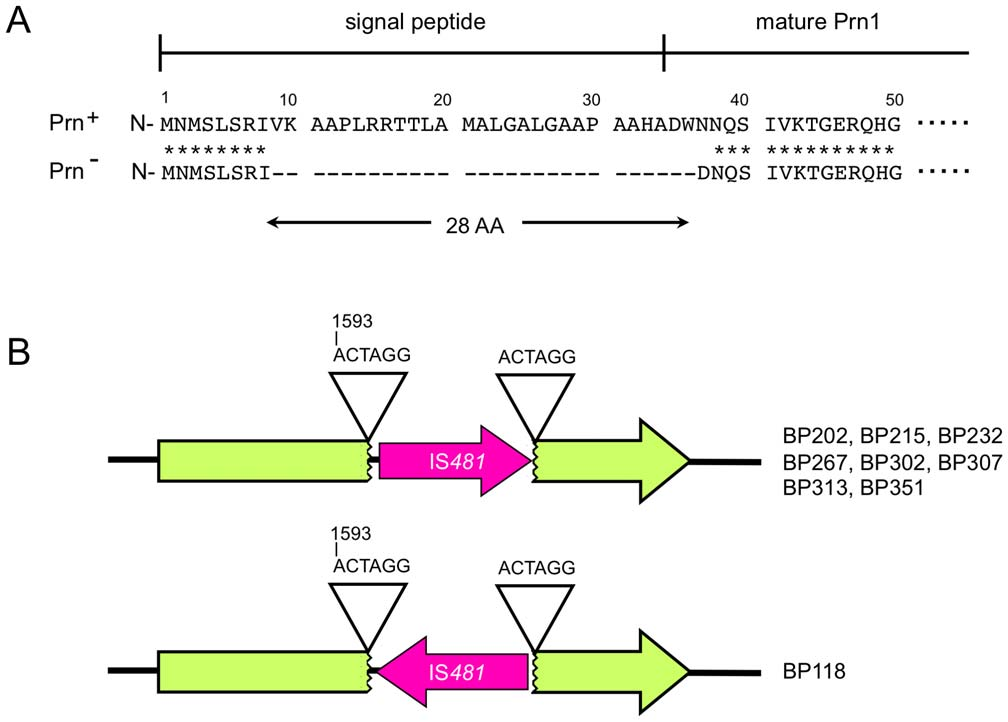
Molecular mechanisms of loss of Prn expression. (A) Deletion of the Prn signal sequence (*prn1*ΔSS). Prn^−^ isolates (*n* = 24) have an 84-bp deletion, resulting in a 28-amino acid deletion (Val^9^ to Trp^36^) in the N-terminal region. (B) IS*481* insertion mutation in Prn1 gene (*prn1*::IS*481*). Eight Prn^−^ isolates have an IS*481* insertion in the forward direction at the 6-bp direct repeats (ACTAGG, 1593–1598 bp) of *prn1*, and 1 isolate had the insertion in the reverse.

To investigate the molecular basis for the loss of Prn expression in Prn^−^ isolates, we sequenced the Prn gene of all 33 Prn^−^ isolates. Two independent mutations were detected, which had caused the loss of Prn1: a deletion of the *prn1* signal sequence (*prn1*ΔSS) and an IS*481* insertion, *prn1*::IS*481* (Figure 2). The *prn1* signal sequence, which plays an important role in localizing Prn to the *B. pertussis* outer cell membrane, was deleted in 24 (73%) out of 33 Prn^−^ isolates. All 24 isolates harboring *prn1*ΔSS had the same 84-bp deletion, resulting in the deletion of 28 amino acid residues (Val^9^–Trp^36^) (Figure 2A). Secondary structure analysis also showed that the deleted DNA sequence is predicted to form a hairpin-loop structure (Figure S1). In contrast, 9 (27%) of 33 Prn^−^ isolates were shown to contain the IS*481* insertion in *prn1*. Eight IS*481* sequences were specifically inserted in the 5′–3′ orientation between a 6-bp direct repeat (ACTAGG, 1593–1598 bp), and 1 was oriented in the opposite direction (Figure 2B).

### Temporal and geographical characterization in Prn^−^ isolates

**Figure 3:**
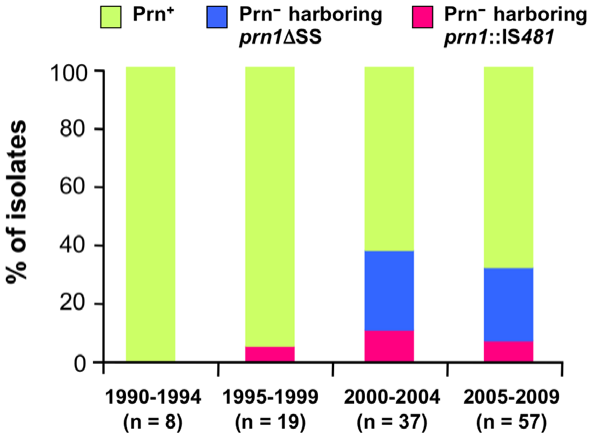
Temporal trend of the occurrence of Prn^−^ isolates in Japan. The frequencies of Prn^−^ isolates harboring *prn1*ΔSS and *prn1*::IS*481* were based on 121 *B. pertussis* isolates collected during 1990–2009. Prn^+^ indicates Prn-expressing isolate.

Figure 3 shows the temporal trend of the frequency of Prn^−^ strains among 121 *B. pertussis* isolates according to the year of collection. The frequencies of Prn^−^ isolate harboring *prn1*ΔSS were 0, 0, 27 and 25% in the periods 1990–1994, 1995–1999, 2000–2004 and 2005–2009, respectively. In contrast, the frequencies of Prn^−^ isolates harboring *prn1*::IS*481* were 0, 5, 11 and 7% in 1990–1994, 1995–1999, 2000–2004 and 2005–2009, respectively. Notably, the total percentage of the Prn^−^ isolates significantly increased from the 2000s, i.e., 0% in 1990–1994, 5% in 1995–1999, 38% in 2000–2004 and 32% in 2005–2009.

**Figure 4:**
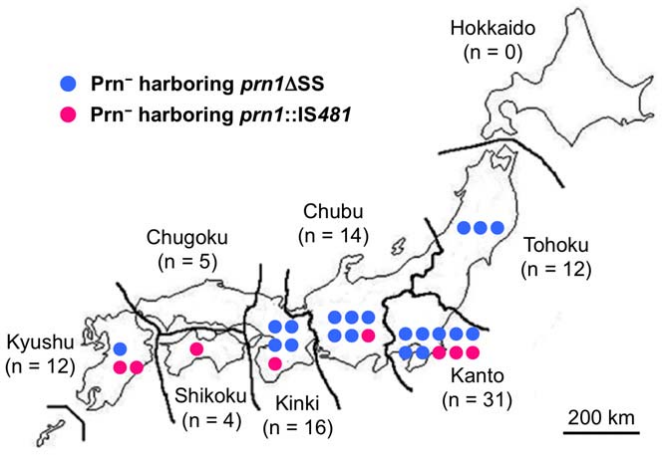
Geographical distribution of Prn^−^ isolates in Japan during 2001–2009. Blue and red circles indicate Prn^−^ isolates harboring *prn1*ΔSS and *prn1*::IS*481*, respectively. Numbers of isolates tested are indicated in parentheses.

During 1990 to 2000, 5 Prn^−^ isolates (*prn1*ΔSS, 4 isolates; *prn1*::IS*481*, 1 isolate) were collected only in the Kanto district. Thereafter, Prn^−^ isolates were collected in several areas during 2001 to 2009 (Figure 4). In the period from 2000 to 2009, 20 Prn^−^ isolates harboring *prn1*ΔSS were collected from Tohoku, Kanto, Chubu, Kinki, and Kyushu districts, and 8 Prn^−^ isolates harboring *prn1*::IS*481* were collected from Kanto, Chubu, Kinki, Shikoku, and Kyushu. These findings indicate that Prn^−^ isolates were present nationwide since 2000.

### Molecular epidemiology of Prn^−^ isolates

**Figure 5:**
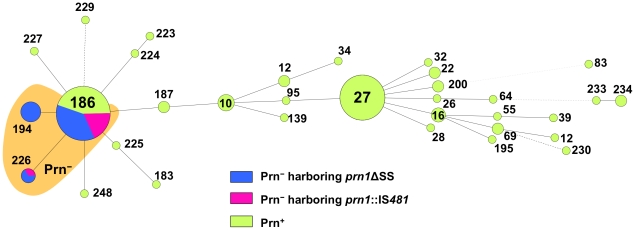
Minimum spanning tree of MLVA of Prn^−^ and Prn^+^ isolates. Total 121 *B. pertussis* isolates, collected during 1990–2009 in Japan, were subjected to MLVA: Prn^−^ isolate harboring *prn1*ΔSS, 24 isolates; Prn^−^ isolate harboring *prn1*::IS*481*, 9 isolates; Prn^+^ isolate, 88 isolates. Each circle in the tree represents a different MLVA type with the MLVA type number. The distance between neighboring genotypes is expressed as the similarity value. Prn^−^ isolates belong to MLVA-186, -194, and -226.

Thirty-three Prn^−^ and 88 Prn^+^ isolates collected by the NIID between 1990 and 2009 were subjected to MLVA. Among the 121 isolates, 33 different MLVA types were identified, of which 10 were novel (MLVA-223 to -227, -229, -230, -233, -234, and -248) (Figure 5 and Table S1). Twenty-six of these MLVA types were present at low frequencies (each, ≤2% of all isolates). Thirty-three Prn^−^ isolates belonged to only 3 MLVA types; 24 isolates (73%) were MLVA-186, 6 isolates (18%) were MLVA-194, and 3 isolates (9%) were MLVA-226. MLVA-186 was the predominant type (frequency, 35% of all isolates), whereas MLVA-194 and MLVA-226 were minor (frequency, 5% and 2%, respectively). The 3 MLVA types were closely related phylogenetically. When categorized by their mutations, 24 Prn^−^ isolates harboring *prn*ΔSS belonged to MLVA-186 (*n* = 16), MLVA-194 (*n* = 6), and MLVA-226 (*n* = 2); 9 Prn^−^ isolates harboring *prn1*::IS*481* belonged to MLVA-186 (*n* = 8) and MLVA-226 (*n* = 1). Thus, MLVA-186 and MLVA-226 were common to both the Prn^−^ isolates, whereas only the Prn^−^ isolate harboring *prn1*ΔSS was typed as MLVA-194.

### Growth advantage of Prn^−^ isolates

We investigated the growth advantage of Prn^−^ isolates by an in vitro growth competition assay. For this purpose, we constructed 2 Prn^+^ back-mutants (Prn^+^-BP59Sm^r^ and Prn^+^-BP202Sm^r^) that were derived from *B. pertussis* isolates BP59 (*prn1*ΔSS) and BP202 (*prn1*::IS*481*), which expressed Prn at a level similar to that of the *B. pertussis* vaccine strain Tohama (Figure S2). *B. pertussis* Tohama produced Prn1 at levels similar to those of other Prn^+^ isolates (Figure 1), indicating that the Prn^+^ back-mutants expressed Prn1 at the same levels as those of naturally occurring Prn^+^ isolates. The Prn^+^ back-mutants also produced PT and FHA at levels equivalent to their parental strains. Moreover, the expression of Fim2 and/or Fim3 was confirmed in the Prn^+^ back-mutants by serotyping (data not shown).

**Figure 6:**
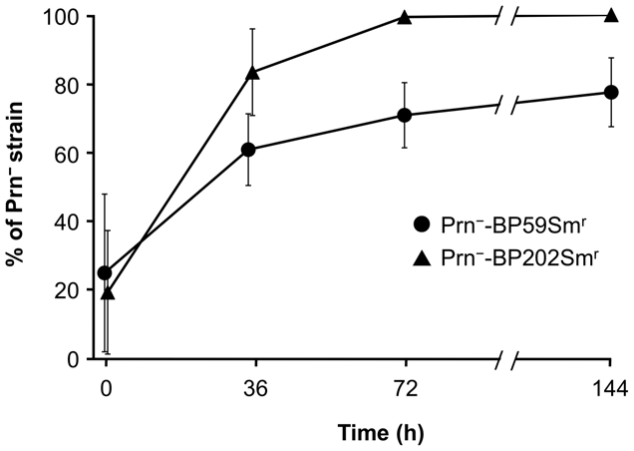
Population dynamics of Prn^−^ strains in in vitro growth competition assay. Prn^+^ back-mutants and parental Prn^−^ strains were mixed in the ratio 4∶1 (Prn^+^-BP59Sm^r^ versus BP59Sm^r^ or Prn^+^-BP202Sm^r^ versus BP202Sm^r^) and cocultured in mSS broth at 36°C. The bacterial cultures were collected at 0, 36, 72 and 144 h, and plated on CSM agar plates. The representation of Prn^−^ strains among 40 colonies was examined by colony-PCR. Data are means and standard deviations from 3 independent experiments.

Figure 6 shows the growth characteristics of the Prn^−^ strain. When the Prn^+^-BP202Sm^r^ back-mutant was co-cultured with its parent, Prn^−^-BP202Sm^r^, the percentage of Prn^−^-BP202Sm^r^ cells increased markedly with time and then reached 100% after 72 h. Similarly, the percentage of Prn^−^-BP59Sm^r^ increased, reaching 71% and 78% at 72 and 144 h, respectively. These results indicate that Prn^−^ strains have higher growth rates in vitro than their Prn^+^ back-mutants. Surprisingly, when the Prn^−^ and Prn^+^ strains were individually cultured in mSS broth, no significant differences were observed in their growth rates (data not shown). Furthermore, no revertant Prn^−^ strains arising from the Prn^+^ back-mutants were observed under the individual culture conditions.

## Discussion

Here, we demonstrate that *B. pertussis* Prn^−^ isolates, generated by 2 different mutations, *prn*ΔSS and *prn*::IS*481*, have significantly increased in Japan since the early 2000s. The emerging Prn^−^ isolates were found nationwide in the 2000s and were found to specifically harbor the vaccine-type *prn1* allele. The rate of Prn^−^ isolation from 2005 to 2009 was 32% (18/57). We believe that this rate is accurate because we investigated all of the isolates (collected in 2005–2009) present in the NIID strain collections except for epidemiologically related cases. Recently, Prn^−^ mutants were also isolated in France at a rate of 5.6% [20], which is significantly lower than for Japan. Taken together, our findings confirm the high prevalence of Prn^−^ strains in the Japanese *B. pertussis* population and raise the question of the pathogenic role of Prn1 in *B. pertussis* infections.

MLVA analysis revealed that various Prn^−^ isolates have high genetic similarity. The Prn^−^ isolates are mainly of the MLVA type 186. The MLVA type has been found in specific countries, Japan and Hong Kong, China [23]. The data on the geographic distribution of Prn^−^ isolates lend support to our hypothesis that the Prn^−^ isolate harboring *prn1*ΔSS or *prn1*::IS*481* has clonally expanded. Interestingly, 3 of 4 French Prn^−^ isolates have an IS*481* insertion in *prn* at the same position as the Japanese Prn^−^ isolates,and the other French Prn^−^ isolate has a larger *prn* deletion (2.4-kbp) involving the 5′-upstream region and signal sequence [20]. Unlike Japanese Prn^−^ isolates, all French Prn^−^ isolates harbor nonvaccine-type *prn2* alleles, indicating that the Japanese Prn^−^ isolates are genetically distinguishable from the French Prn^−^ isolates.

Japanese Prn^−^ isolates harboring *prn1*ΔSS had an 84-bp deletion in the *prn* signal sequence. The deleted DNA sequence is predicted to form a hairpin loop structure, suggesting that the DNA loop might be excised from the *prn1* by DNA repair enzyme(s) (Figure S1). Although the deleted sequence does not affect the translational reading frame of Prn1 gene, a truncated Prn1 precursor was not detected in the Prn^−^ isolates (Figure 1). Interestingly, in vitro transcriptional-translation analysis revealed that the *prn1*ΔSS gene could be transcribed and translated as a truncated Prn1 precursor (data not shown). This suggests immediate degradation of truncated Prn1 in the bacterial cell. In contrast, Prn^−^ isolates harboring *prn1*::IS*481* were disrupted by an IS*481*-insertion at a 6-bp direct repeat (ACTAGG, 1593–1598 bp) in *prn1*. The direct repeats in *prn1* conform to the recognition sequence of IS*481*, NCTAGN [24]. IS*481* is present in multiple copies in the *B. pertussis* chromosome and the number of lost genes increased with time by IS*481*-dependent rearrangement [22], [25]. Taken together with information published on French Prn^−^ isolates, 3 different mechanisms, IS*481* insertion and 2 *prn* deletions (84-bp and 2.4-kb), have contributed to the loss of Prn expression in *B. pertussis*. These gene disruptions strongly suggest that human host factors (genetic factors and immune status) that select for Prn^−^ strains have arisen.

Prn's adhesin properties have been investigated both in vitro and in vivo [26], [27], [28], [29]. A recent study showed that *B. pertussis* Prn^−^ mutants colonized less well than Prn^+^ strains in mice [18]. It is also known that Prn prevents either bacterial adherence or internalization or both, to human monocyte-derived dendritic cells [30] and that it also plays a role in resistance to neutrophil-mediated clearance [31]. Further, *B. bronchiseptica* Prn is required for optimal colonization of the swine respiratory tract [32]. Prn may therefore play a crucial role in bacterial adhesion and in survival and colonization in humans. However, here we have discovered a high prevalence of Prn^−^ isolates in recent *B. pertussis* populations in Japan. This observation strongly suggests that loss of Prn does not significantly reduce bacterial fitness in the present environment. Prn is highly conserved among the *Bordetella* species. Surprisingly, Prn^−^ isolates of the human pathogen *Bordetella parapertussis* have also been found recently in France [33]. This finding supports our hypothesis that the role of Prn in fitness (or transmission) has diminished in some hosts.

We demonstrate here that Prn^−^ strains have a higher growth potential than their Prn^+^ back-mutants in vitro (Figure 6). The increased growth advantage of Prn^−^ strains provides knowledge about their biological properties. The most likely explanation for prevalence of Prn^−^ strains is vaccine-driven selection. Prn is an important antigenic component of most current aP vaccines, and it plays a role in eliciting protective immunity [4], [6], [7], [34], [35], leading to the suggestion that Prn^−^ strains have escaped the immune response to Prn. The herd immunity by aP vaccines could exert selective pressure for pathogen evolution, like the emergence of the PT promoter (*ptxP3*) lineage that produces higher levels of PT [36]. In fact, Prn1 strains might be more fit in unvaccinated than in vaccinated populations [18]. In Japan, four currently used vaccines are produced from *B. pertussis* vaccine strain Tohama; two vaccines contain Prn1 and others do not contain it [3]. The aP vaccines that can be used interchangeably for routine immunization of infants have been introduced in Japan since 1981. Subsequently, Prn1 clinical strains have been gradually replaced by Prn2 strains since the mid-1990s [17], and Prn^−^ strains significantly increased since the early 2000s (as shown here). These observations suggest the interesting possibility that Prn^−^ strains may have increased fitness in vaccinated populations, i.e., Prn1 strains are most affected by vaccination with aP vaccines containing Prn1, whereas Prn2 strains producing non-vaccine type Prn are not. However, in the present study, the vaccination status of the majority of patients infected with Prn^−^ strain was unknown (Table S1). Thus, the relationship between Prn^−^ strains and vaccine efficacy is currently unclear. Further studies now underway on patients' background are needed to verify the hypothesis.

In conclusion, Prn^−^ strains have significantly increased in *B. pertussis* populations since the early 2000s in Japan. *B. pertussis* Prn^−^ strains have also been found in France, as well as among isolates of the human pathogen *B. parapertussis*. These observations suggest that Prn expression may be not essential for fitness of *Bordetella* species in the recent host environment and that Prn^−^ strains may be fit in humans immunized with aP vaccines. Further analyses and global surveillance are required to elucidate the emergence of Prn^−^ strains.

## Materials and Methods

### Bacterial strains

We studied 121 *B. pertussis* clinical isolates collected from 1990 to 2009 in Japan (Table S1). The isolates were selected from the National Institute of Infectious Diseases (NIID) strain collections to reflect the same temporal distribution of the *prn* allele [17], [37]. Seventy-nine isolates harbor the vaccine-type *prn1* allele, and 41 and 1 isolates harbor nonvaccine-type *prn2* and *prn9* allele, respectively. All the isolates were epidemiologically unrelated cases of pertussis. The isolates were cultured on Bordet-Gengou agar (Difco) supplemented with 1% glycerol and 15% defibrinated horse blood and incubated at 36°C for 2–3 days.

### Immunoblotting and serotyping

*B. pertussis* isolates were subcultured on cyclodextrin solid medium (CSM) [38]. Total protein was extracted from bacterial cells with SDS-lysis buffer (62.5 mM Tris-HCl, 1% SDS, 10% glycerol, 5% 2-mercaptoethanol, pH 6.8). Protein samples (1 µg protein) were subjected to 10% SDS-PAGE, transferred to nitrocellulose membranes (Bio-Rad), and incubated with anti-Prn1, anti-FHA, or anti-PT antiserum. Antigen-antibody complexes were visualized using horseradish peroxidase (HRP)-conjugated secondary antibody (Bio-Rad) and ECL Western Blotting Detection Reagents (GE Healthcare) and the blots imaged using a LAS-3000 (Fujifilm, Tokyo, Japan). The anti-Prn1 antiserum was generated in mice with purified Prn1 derived from *B. pertussis* strain Tohama.

Serotyping of *B. pertussis* isolates was performed in a microplate agglutination assay using ati-Fim2 and anti-Fim3 monoclonal antibodies [39]. The anti-Fim2 (NIBSC 04/154) and anti- Fim3 (NIBSC 04/156) antibodies were obtained from the National Institute for Biological Standard and Control. *B. pertussis* strain 18323 expressing both Fim2 and Fim3 was used as a positive control [40].

### DNA sequencing

DNA sequencing of PCR fragments representing relevant regions of *prn* was performed as described [14], [41]. Sequence reactions were carried out with a BigDye® Terminator v3.1 Cycle Sequencing Kit (Applied Biosystems), and the products were sequenced using an ABI PRISM 3130xl Genetic Analyzer (Applied Biosystems). The complete open reading frames of all Prn^−^ isolates (*n* = 33) were determined.

### MLVA

MLVA typing was performed as described previously [23], [42]. Six variable-number tandem-repeat loci (VNTR1, 3a, 3b, 4, 5, and 6) were amplified by PCR, and the fragments were separated using an ABI PRISM 3130xl Genetic Analyzer with GeneScan™-600LIZ® (Applied Biosystems) as an internal lane size standard. For each VNTR locus, the size of the PCR product was converted to a number of repeat units as alleles using GeneMapper software ver.4.0 (Applied Biosystems). Each MLVA type was assigned as described earlier [23], [43], and novel MLVA type numbers were assigned by Dr. F. Mooi, Netherlands Centre for Infectious Diseases Control, National Institute for Public Health and the Environment, The Netherlands.

Minimum spanning trees were generated from the 6 MLVA loci using the FPQuest Software (Bio-Rad). Links were generated between MLVA types with a categorical comparison algorithm, with the following rules in priority order: (1) Link types must have the maximum number of single-locus variants (SLVs), (2) types must have the maximum number of SLVs and double-locus variants, and (3) types must have the maximum number of entries.

### Generation of Prn^+^ back-mutants

Two Prn^+^ back-mutants (Prn^+^-BP59Sm^r^ and Prn^+^-BP202Sm^r^) were constructed from Prn^−^ isolates BP59 (*prn1*ΔSS) and BP202 (*prn1*::IS*481*) by double cross-over homologous recombination, respectively [44]. To construct the Prn^+^-BP59Sm^r^ back-mutant, a 2.4-kbp DNA fragment (prnA) encoding the intact *prn* signal sequence was amplified by PCR with attB1-sigF and attB2-sigR primers (Table S2) using *B. pertussis* Tohama genomic DNA as the template. The resulting PCR product was cloned into pDONR221 to obtain pDONR-prnA using the adaptor PCR method in the Gateway cloning system (Invitrogen). The pDONR-prnA and pABB-CRS2 [45] were combined to obtain pABB-prnA using the Gateway cloning system. pABB-prnA was introduced into *E. coli* SM10λ*pir* and transconjugated into strain BP59Sm^r^ (streptomycin-resistant, Sm^r^). The resulting mutant was designated Prn^+^-BP59Sm^r^.

To construct the Prn^+^-BP202Sm^r^ back-mutant, a 2.3-kbp DNA fragment (prnB) encoding intact *prn* gene was PCR-amplified using attB1-ISF and attB2-ISR primers (Table S2). Plasmid pABB-prnB was constructed from pDONR-prnB and then transconjugated into strain BP202Sm^r^ via *E. coli* SM10λ*pir*. The resulting mutant was designated Prn^+^-BP202Sm^r^.

To confirm site-specific recombination, the *prn* of Prn^+^ back-mutants was sequenced, confirming that the pABB vector sequence was entirely removed from *prn* of both Prn^+^ back-mutants.

### In vitro growth competition assay

Prn^+^ back-mutants (Prn^+^-BP59Sm^r^ and Prn^+^-BP202Sm^r^) and their parental Prn^−^ strains (BP59Sm^r^ and BP202Sm^r^) were inoculated into modified Stainer-Scholte (mSS) broth [46], and cultured with shaking at 36°C. After 24 h, the culture solutions were diluted to an optical density (650 nm) of 0.2 with mSS broth. The Prn^+^ back-mutant (2.4 ml) and its parental Prn^−^ strain (0.6 ml) were mixed at the ratio 4∶1 and co-cultured with shaking at 36°C. The bacterial cultures (30 µl) were subcultured once in fresh mSS broth (3 ml) for 72 h.

The bacterial cultures were collected at 0, 36, 72, and 144 h, diluted with 1% casamino acid solution containing 0.6% NaCl, pH 7.1, and plated on CSM agar plates. After incubation for 3–4 days, 40 colonies were checked for *prn* size by colony-PCR performed as follows: 94°C for 2 min; 30 cycles of 98°C for 10 s, 55°C for 30 s, and 68°C for 3 min; and final incubation at 72°C for 5 min. Primer sets, PrnF and 1053R, and PrnF and PrnR, were used for strains BP59Sm^r^ (*prn1*ΔSS) and BP202Sm^r^ (*prn1*::IS*481*), respectively (Table S2).

### Nucleotide sequence accession number

The nucleotide sequence data reported in this study have been deposited in the DDBJ/EMBL/GenBank nucleotide sequence databases under accession numbers AB670735 to AB670737.

## Supporting Information

Figure S1 **A hairpin loop structure in the signal sequence (SS) of Prn gene.** Twenty-four Prn^−^ isolates harboring *prn1*ΔSS have an 84-bp deletion at position 26–109 bp, corresponding to the hairpin loop. The schematic shows a simplified map.
(TIF)

Figure S2 **Expression of Prn, PT, and FHA in Prn^+^ back-mutants derived from Prn^−^ isolates.** Prn^+^ back-mutants (Prn^+^-BP59Sm^r^ and Prn^+^-BP202Sm^r^) were constructed from streptomycin-resistant Prn^−^ isolates, BP59Sm^r^ (*prn1*ΔSS), and BP202Sm^r^ (*prn1*::IS*481*), respectively. Total protein (1 µg) extracted from the bacterial cells was subjected to SDS-PAGE and analyzed by immunoblotting with anti-Prn1, anti-PT or anti-FHA antiserum. Total protein (1 µg) from *B. pertussis* Tohama was run on the gel as a positive control. PT-S1 indicates the S1 subunit of PT.
(TIF)

Table S1 **Characteristics of** ***B. pertussis*** **isolates.**
(XLSX)

Table S2 **PCR primers in this study.**
(XLSX)
