# Supplementary figures and images for: Prevalence and Genetic Characterization of Pertactin-Deficient Bordetella pertussis in Japan
Source: PLoS One. 2012 Feb 14;7(2):e31985. doi: 10.1371/journal.pone.0031985 (PMC3279416; doi:10.1371/journal.pone.0031985)

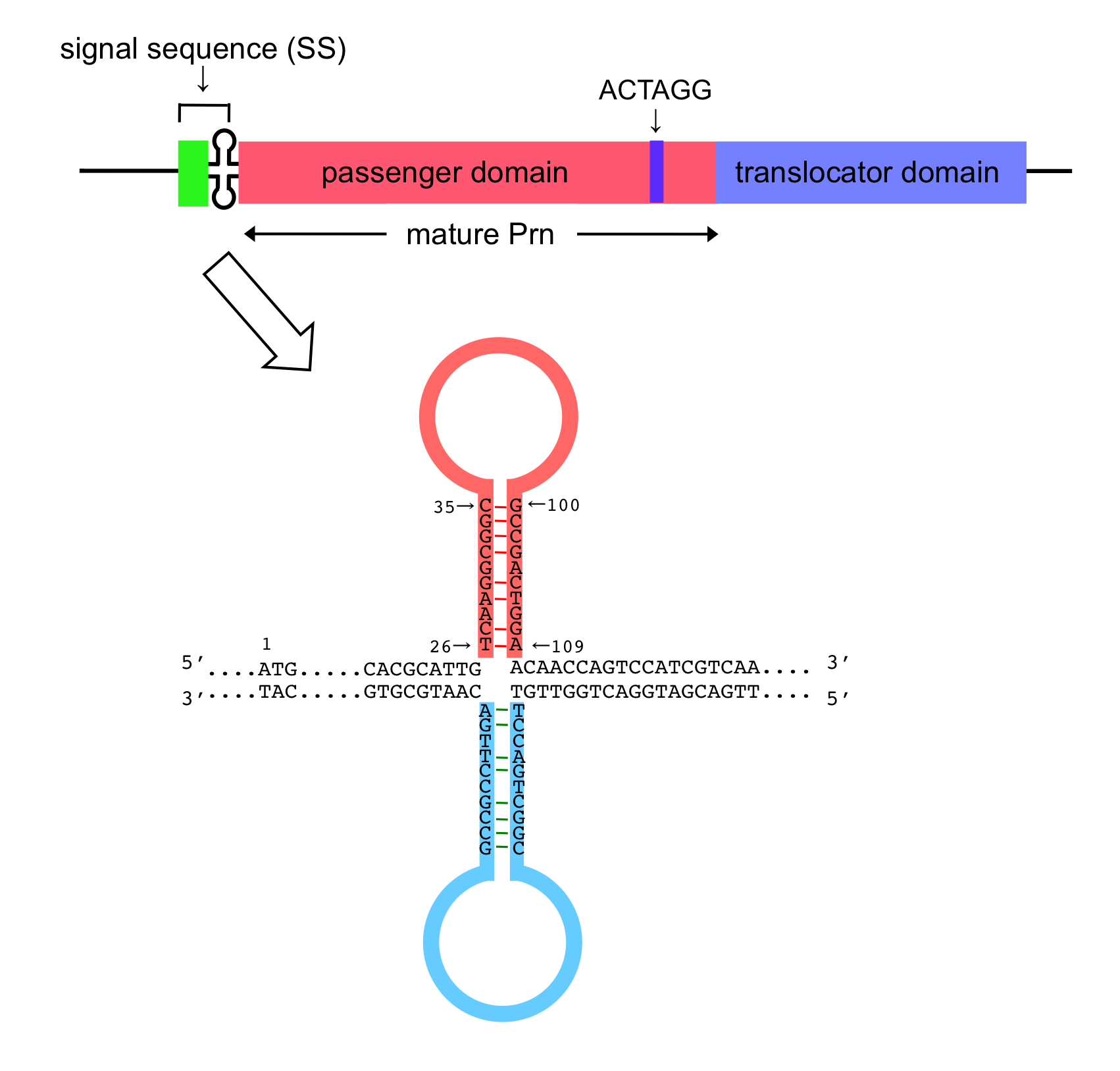

Supplement: Figure S1 — A hairpin loop structure in the signal sequence (SS) of Prn gene. Twenty-four Prn− isolates harboring prn1ΔSS have an 84-bp deletion at position 26–109 bp, corresponding to the hairpin loop. The schematic shows a simplified map. (TIF) [file pone.0031985.s001.tif]

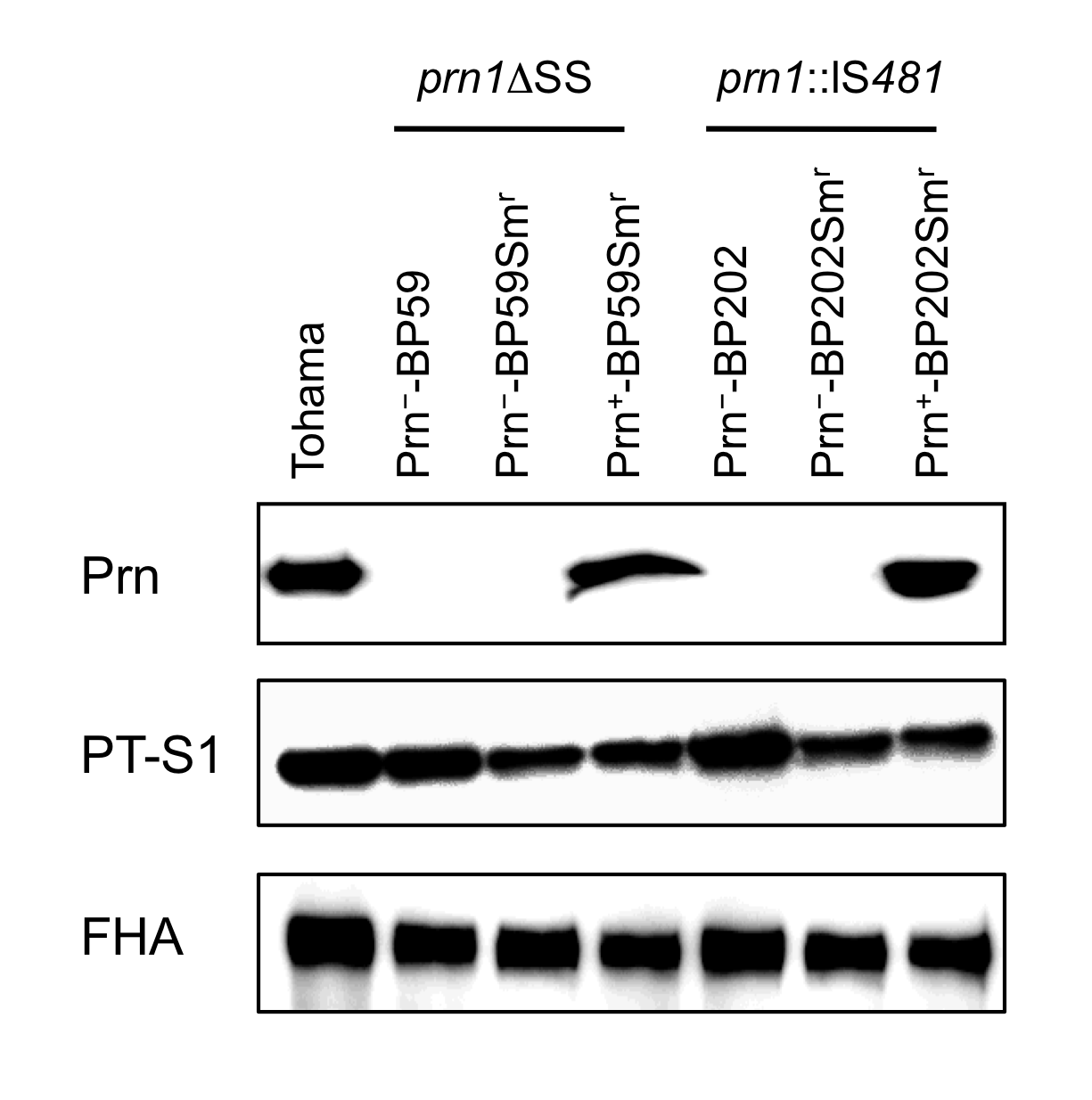

Supplement: Figure S2 — Expression of Prn, PT, and FHA in Prn+ back-mutants derived from Prn− isolates. Prn+ back-mutants (Prn+-BP59Smr and Prn+-BP202Smr) were constructed from streptomycin-resistant Prn− isolates, BP59Smr (prn1ΔSS), and BP202Smr (prn1::IS481), respectively. Total protein (1 µg) extracted from the bacterial cells was subjected to SDS-PAGE and analyzed by immunoblotting with anti-Prn1, anti-PT or anti-FHA antiserum. Total protein (1 µg) from B. pertussis Tohama was run on the gel as a positive control. PT-S1 indicates the S1 subunit of PT. (TIF) [file pone.0031985.s002.tif]
